# Systemic zoledronate treatment both prevents resorption of allograft bone and increases the retention of new formed bone during revascularization and remodelling. A bone chamber study in rats

**DOI:** 10.1186/1471-2474-7-63

**Published:** 2006-08-04

**Authors:** Jörgen Åstrand, Anna Kajsa Harding, Per Aspenberg, Magnus Tägil

**Affiliations:** 1 Department of Orthopaedics, Lund University Hospital, SE-221 85 Lund, Sweden; 2 Department of Neuroscience and Locomotion, Faculty of Health Sciences, Division for Orthopaedics and Sports Medicine, Linköping University, Linköping, Sweden

## Abstract

**Background:**

In osteonecrosis the vascular supply of the bone is interrupted and the living cells die. The inorganic mineral network remains intact until ingrowing blood vessels invade the graft. Accompanying osteoclasts start to resorb the bone trabeculae and gradually replace the bone. If the osteonecrosis occurs in mechanically loaded parts, like in the subchondral bone of a loaded joint, the remodelling might lead to a weakening of the bone and, in consequence to a joint collapse. Systemic bisphosphonate treatment can reduce the resorption of necrotic bone. In the present study we investigate if zoledronate, the most potent of the commercially available bisphosphonates, can be used to reduce the amount or speed of bone graft remodeling.

**Methods:**

Bone grafts were harvested and placed in a bone chamber inserted into the tibia of a rat. Host tissue could grow into the graft through openings in the chamber. Weekly injections with 1.05 μg zoledronate or saline were given subcutaneously until the rats were harvested after 6 weeks. The specimens were fixed, cut and stained with haematoxylin/eosin and used for histologic and histomorphometric analyses.

**Results:**

By histology, the control specimens were almost totally resorbed in the remodeled area and the graft replaced by bone marrow. In the zoledronate treated specimens, both the old graft and new-formed bone remained and the graft trabeculas were lined with new bone. By histomorphometry, the total amount of bone (graft+ new bone) within the remodelled area was 35 % (SD 13) in the zoledronate treated grafts and 19 % (SD 12) in the controls (p = 0.001). Also the amount of new bone was increased in the treated specimens (22 %, SD 7) compared to the controls (14 %, SD 9, p = 0.032).

**Conclusion:**

We show that zoledronate can be used to decrease the resorption of both old graft and new-formed bone during bone graft remodelling. This might be useful in bone grafting procedure but also in other orthopedic conditions, both where necrotic bone has to be remodelled i.e. after osteonecrosis of the knee and hip and in Perthes disease, or in high load, high turnover conditions like delayed union, periprosthetic osteolysis or bone lengthening operations. In our model an increased net formation of new bone was found which probably reflects that new bone formed was retained by the action of the bisphosphonates rather than a true anabolic effect.

## Background

Osteonecrosis is hypothesised to be caused by insufficient circulation [1]. It can occur after trauma or be the result of other events or conditions that compromise the circulation, such as corticosteroid treatment, scuba diving, sickle cell anemia, alcoholism and pregnancy [2]. Necrotic bone retains its load bearing capacity [3], but as revascularization and remodelling starts, resorption and bone forming will occur simultaneously. In mechanically loaded parts, like in the subchondral bone of a loaded joint, the remodelling might lead to a weakening of the bone and, in consequence, to a joint collapse [14,5,6]. In consequence, it is not the death of bone cells per se that causes structural failure, but rather, the resorption of necrotic bone and the imbalance between formation and resorption.

Resorption is mediated by osteoclasts, recruited from their hematopoetic origin, and occurs during or following the revascularization of the necrotic area. Osteoclastic activity can be reduced with bisphosphonates, a class of drugs in clinical use for the treatment of osteoporosis, Paget's disease and osteolytic metastases. Circulating bisphosphonates will bind to the bone mineral. When bone is resorbed by osteoclasts, bisphosphonates are internalized by the cell and interfere with cell metabolism leading to apoptosis of the osteoclast [7]. Systemic bisphosphonate treatment can thus reduce the resorption of necrotic bone and is well established for treatment of tumour metastases and osteoporosis. Lately, several other applications of the bisphosphonates have been proposed in the orthopaedic practice, for example as treatment to reduce the risk of structural failure and joint surface collapse after osteonecrosis of the hip in children after SCFE and Perthes [8] and in the adult [9-11], to prevent the collapse in Charcot feet [12], to decrease prosthetic migration [13] and periprosthetic osteolysis in hip replacements [14]and to increase the strength of the regenerate in bone lengthening [15] or bone grafting procedures [16].

Zoledronate is a new and more potent biphosphonate, which can, just as previously shown with alendronate [17]decrease the bone resorption during graft remodelling but has the advantage of being more potent. Compared to other bisphosphonates it can therefore be administered less frequently, and in treatment of osteoporosis as seldom as once a year [18]. In the present study we investigate if zoledronate can be used to reduce bone graft remodelling and if the time span between the doses can be prolonged.

## Methods

**Figure 1:**
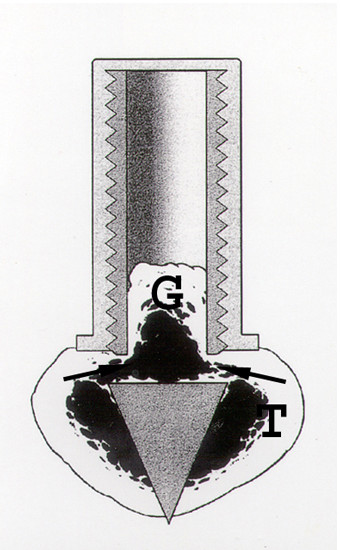
The bone conduction chamber in situ in the proximal tibia (T). The graft (G) is placed in the chamber and mesenchymal tissue grows in from the bottom upwards into the bone graft, which subsequently remodels. Arrows point at ingrowth openings. (Reproduced with permission from Eur J Exp Musculoskel Res 2: 70, 1993).

We used a model with a cancellous graft in a *bone conduction chamber *(BCC, Fig 1; [19]). The chamber is basically a threaded titanium cylinder, made of two half cylinders held together by a hexagonal screw cap. The interior of the chamber is 7 mm long and has a diameter of 2 mm. One end of the implant is screwed into the proximal tibia of a rat. At this end there are two ingrowth openings where tissue can grow in from the subcortical bone into a graft placed in the chamber.

### Grafts

Ten donor rats (female ca 200 g) were killed by an overdose of pentobarbital and bone grafts harvested from the proximal tibias. The epiphyses and the growth plates were discarded to remove the cartilage. A cylindrical cancellous bone rod was taken out from each tibia in the axial direction, using a hand-held hole cutter, and frozen at -70C for a week. At surgery, the grafts were thawed and placed in the chambers, which then were inserted into the right leg of the recipient animals.

### Surgical procedure

Twenty male Sprague-Dawley rats (382 – 425 grams, Møllegaard, Copenhagen, Denmark) served as graft recipients. They were kept in animal facilities for 1 week before experiments started (22°C; two rats in each cage, free access to food pellets and water). The rats were anesthetized with peritoneal injections of 0.6 to 0.7 mL of a solution containing pentobarbital (15 mg/mL) and diazepam (2.5 mg/mL)

Under aseptic conditions, longitudinal incisions were made bilaterally over the antero medial aspect of the proximal tibial metaphyses. After incising and raising the periosteum, the medial and posterior lateral cortices were pierced with a 1 mm spike just anterior to the insertion of the medial collateral ligament. The hole created in the medial cortex was enlarged manually with a 2.7 mm drill. The chambers were then screwed into position so that the bone ingrowth openings were placed at the level of the cortical bone, and the pointed end of the implant penetrated the opposite cortical bone. The wound was closed leaving the entire chamber subcutaneous, palpable through the skin but with its ingrowth openings situated subcortically. Local anaesthetics and postoperative buprenorfin was given for pain relief.

### Injections

Postoperatively, 0.5 ml subcutaneous injections with 1.05 μg zoledronate were given at day 4 and then weekly until harvest. The controls were given the same amount of saline solution at the same regime.

### Evaluation

The chambers were harvested after 6 weeks. The specimens were fixed in 4 % formalin, decalcified, embedded in paraffin, cut parallel to the long axis of the chamber with a microtome and stained with haematoxylin and eosin. Three sections from each specimen, each at 300 μm distance from the other, and showing the entire chamber contents, were used for histological and histomorphometric analyses. All slides were investigated in random order.

The area of the new ingrown bone was measured by circumscribing it on a digitizing table using the Videoplan™ equipment (Kontron Bildanalyse GmBH, Esching, Germany) at 20× screen magnification. This area includes marrow cavities and graft remnants that had been surrounded by new bone. The mean bone ingrowth distance in each slide was calculated by dividing the new bone area with the distance between the walls of the chamber.

The bone density in the remodelled area was evaluated using point counting and a Merz grid at 40x screen magnification. Using an ocular with 36 crossing lines, the frequency of the crossings covering bone tissue was recorded. In each 6 randomly chosen areas were analysed. The frequencies then were then expressed as a percentage of the total bone area. In each graft six randomly chosen areas were measured in the mid part and the ends. The distinction between living and dead bone was based on matrix staining, presence of osteocytes and trabecular shape. For all measurements the data were averaged to form a single value for each animal. The results were tested for significance using Student's T-test. The study was approved by the local ethical committee (M 44-03).

## Results

**Figure 2:**
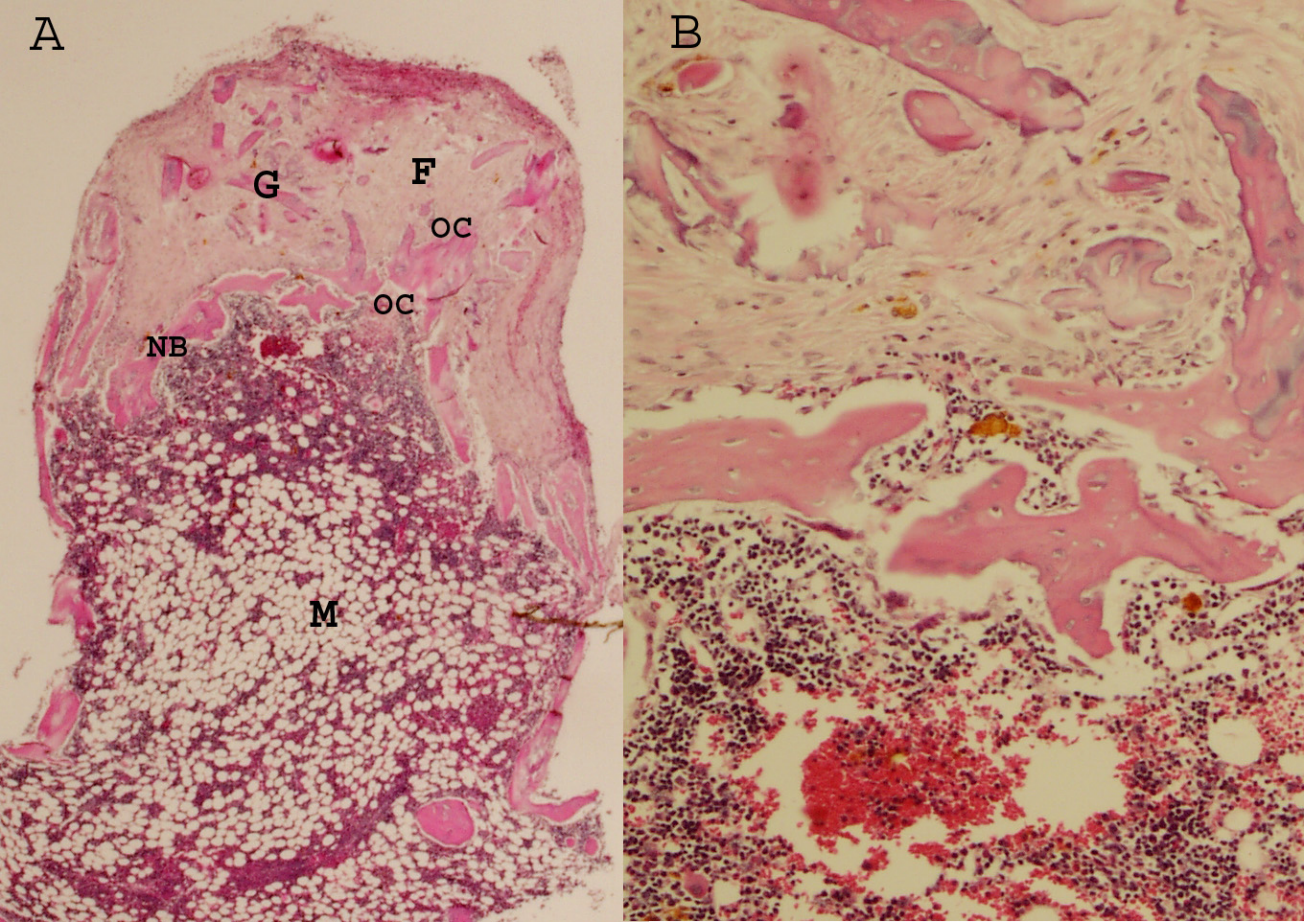
**A **Untreated control graft after 6 weeks in the chamber Ingrowth from openings in the bottom of the chamber and upwards. At the top a vascularized fibrotic tissue has advanced furthest into the graft. The fibrotic tissue(F) is revascularized but the graft trabeculas (G), have not remodelled or started to resorb. The frontier of new-formed bone (NB) advances as a thin rim into the graft, preceeded by osteoclasts (OC). Since the graft is remodelling within a stress-shielded titanium chamber, without any deformation of the tissue, the new-formed bone resorb immediately. Below the new bone, therefore a marrow cavity (M) is formed where both the new-formed as well as the old graft bone is resorbed. (Hematoxylin Eosin × 20). **B**. Magnification of the borderzone between remodelled and resorbed bon, the front of new formed bone (Hematoxylin Eosin × 100)

**Figure 3:**
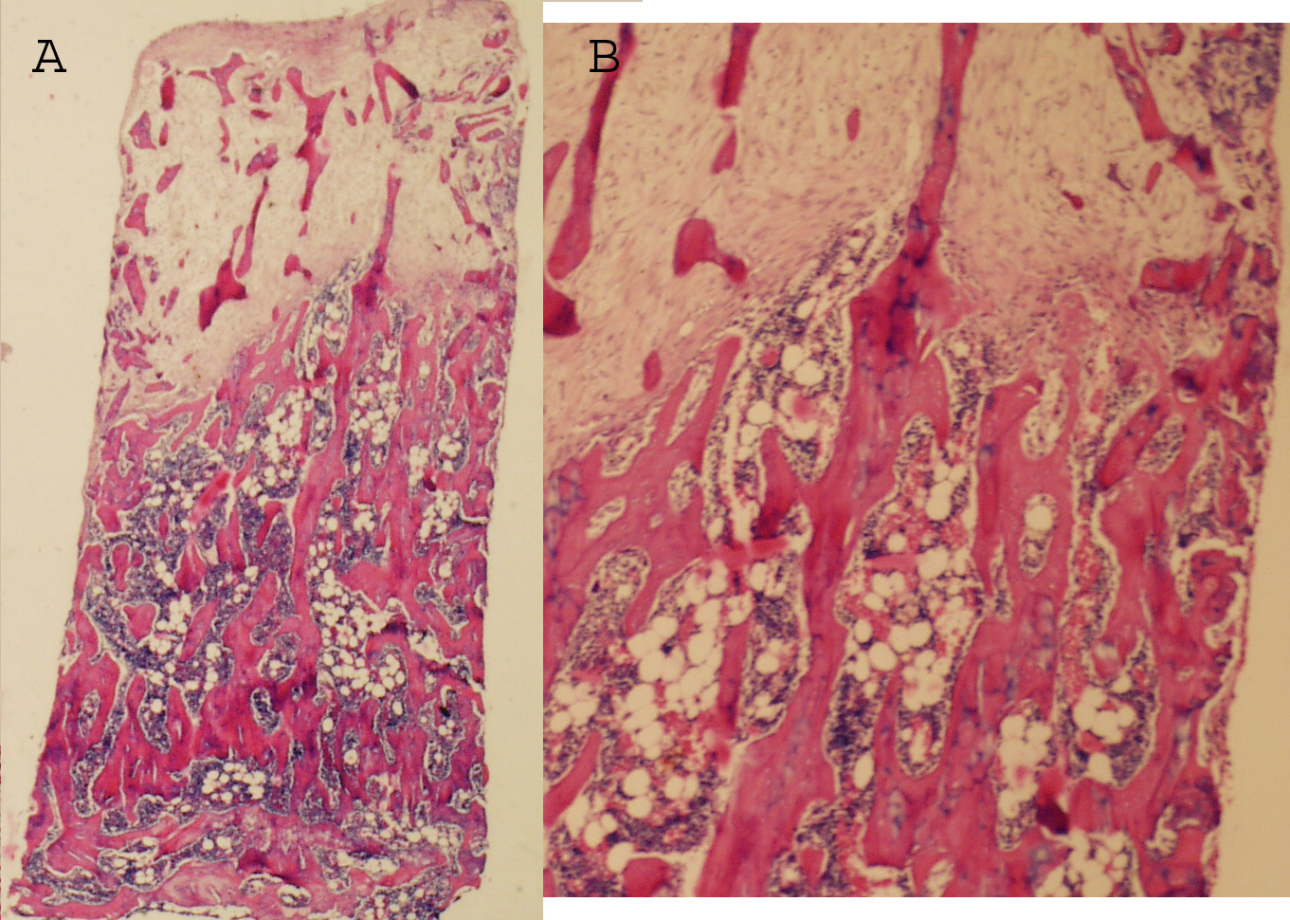
**A **Zoledronate treated graft after 6 weeks. No marrow cavity has formed behind the ossification front but the graft trabeculas below are still remaining to a large extent. The total amount of bone, both newly formed and remaining graft bone is higher in the remodelled area compared to the control. (Hematoxylin Eosin × 20). **B **Magnification of the borderzone with retained trabeculas in the remodelled area.

No infection occurred. One rat died directly postoperatively of unknown cause. In all specimens vascularised soft tissue (Fig 2 and 3) had invaded the whole grafts forming a fibrotic marrow at the upper part of the specimen. This area was revitalized and revascularised but without any signs of bone remodelling. The total distance of the soft tissue ingrowth, which corresponds to the revascularised parts of the graft, did not differ between treated and controls (4.24 mm, SD 0.90 and 3.90 mm, SD 1.01 mm, p = 0.45).

Further down in the specimens, roughly halfway through the graft, a zone with active new bone formation was seen closely preceded by resorption of the graft. This new bone ingrowth frontier marks the border of bone remodelling where a primary bone formation occurs. A tendency towards an increased ingrowth distance was found in the zoledronate treated grafts (2.65 mm, SD 0.48 and 2.12 mm, SD 0.63, p = 0.052).

In the controls, this frontier of active bone formation was thin and both the graft and the new formed bone *underneath *this primarily formed bone appeared to be immediately resorbed and replaced by a haematogenous or fatty bone marrow (Fig 2). In contrast, in the grafts in rats treated with zoledronate, the graft and new-formed bone underneath the active bone formation front remained intact with new bone lining the graft trabeculae, leaving only little space for the marrow (Fig 3). Measuring the bone density in the remodelled bone, i.e. both the new bone-forming frontier as well as the more or less remodelled area underneath this front, the total bone volume fraction within the *remodelled *area was 35% (sd13) in the zoledronate treated grafts compared to 19% (sd 12) in the controls (p = 0.01). Dividing the total amount of bone into new formed and remaining graft bone, the total amount of retained graft bone was 13% (sd 6) for the zoledronate treated specimens compared to 5% (sd 5) in the controls. Also the amount of new bone was increased in the treated specimens compared to the controls and the proportion of new-formed bone within the remodelled area was 22% (sd 7) in the zoledronate treated grafts and 14 % (sd 9) in the controls (p = 0.03).

## Discussion

Previously, we have shown alendronate to be effective in preventing or delaying the resorption of a bone graft [17]In the present study we show that zoledronate, a more potent bisphosphonate is equally effective in weekly injections as the three times a week alendronate treatment. The bone chamber model is a stress-shielded model where the resorptive stimulus is high. Normally, all graft and new formed bone is resorbed as the remodelling is finished and an anticatabolic drug such as a bisphosphonate is probably more effective than in a mechanically loaded environment. In our experiments, we use subcutaneous injections, which could mean that less bisphosphonate is entering the systemic circulation. With higher doses or intravenous administration, this could hypothetically constitute a negative influence on bone formation. Further, a prolonged effect of the drug with slow-release effects could be the effect of a subcutaneous deposition compared to an intravenous administration.

Allografts were used as a model for necrotic, autologous bone. The strain of Sprague-Dawley rats used for this study is, however, inbred to such an extent that no differences can be detected in the incorporation of auto- versus allografts in this model [20]. Theoretically, it could be assumed that also after freezing and thawing, allografts are more immunogenic than autologous bone and if so, allografts would stimulate bone resorption even more than an autograft. Since we wanted to study whether it is possible to reduce bone resorption during revascularization, such an increased tendency to resorption would necessitate an even greater protective effect of zoledronate in this model.

Lately, a number of new indications have been proposed for orthopaedic applications of bisphosphonates, relating to the ability of the substance to decrease the resorption of necrotic bone during revascularization and remodelling after an avascular necrosis. It appears that bone under remodelling can retain its form better if the remodelling is slowed down by for example a bisphosphonate [4-6]. Still, there are concerns that bisphosphonates might interfere with the normal fracture and bone healing [21]. Theoretically, the bone stock can be negatively influenced, both in the long run, by decreasing the ability to maintain and repair microfractures over years, but also in the short term by interfering with the bone formation. Osteoclasts are necessary for bone formation by the osteoblasts. Regarding the concern for long time complications, long time follow-up data regarding alendronate does not show an increased fracture rate in patients on osteoporosis prophylaxis treatment for up to 10 year [22]. Zoledronate, however, is more potent than alendronate and could hypothetically interfere more, but no corresponding long time data exist. Regarding the fear to use a bisphosphonate for the short term influence on fracture healing, the results of the present and other recent experiments, using bone chambers as a model for remodelling of a graft or necrotic bone show, not only a decreased resorption as expected, but also an increased amount of new bone in the remodelled area. Although non-significant, a tendency towards an increased bone ingrowth distance or speed was noticed. One could interpret these results as if bisphosphonates would be not be merely an anticatabolic, but also function as an anabolic substance as hypothesized in some in vitro [23,24] and in vivo studies [25]. Bisphosphonates are for example found to have an antiapoptotic effect in osteocytes and osteoblasts [26], but several other explanations to the findings of an increased amount of newly formed bone can, however, be discussed. In contrast to cortical bone, remodelling in cancellous bone does not require cutting cones to make space for new bone. In cancellous bone there is sufficient space for new bone to form and often the new bone formation appears as appositional growth, covering the dead bone graft, which is not resorbed because it is bisphosphonate treated. In consequence, a larger surface area exists to lay down new forming bone onto (Fig 3). The fact that we find an increased amount of newly formed bone does not necessarily mean that more bone has formed or that zoledronate by any means is anabolic. New-formed bone might simply just prevail for a longer period if bone resorption is reduced. With the bisphosphonates, most of the calcified tissue that was present or has formed since the remodelling of the graft started will remain, both the old graft bone as well as the newly formed bone growing into the graft.

Regardless of reason, bone formation during bone graft remodelling was not decreased when treated with bisphosphonate in our study. On the contrary, more bone was found within the remodelled graft, both old graft and newly formed. Bisphosphonate treatment might, however, erroneously mimic an increased new bone formation, due to the absent resorption of the new formed bone.

## Conclusion

We show that zoledronate can be used to decrease the resorption of both old graft and new-formed bone during bone graft remodelling. This might be useful in bone grafting procedure but also in other orthopedic conditions, both where necrotic bone has to be remodelled i.e. after osteonecrosis of the knee and hip and in Perthes disease, or in high load, high turnover conditions like delayed union, periprosthetic osteolysis or bone lengthening operations. In our model an increased net formation of new bone was found which probably reflects that new formed was retained by the action of the bisphosphonates rather than a true anabolic effect.

## Competing interests

'The author(s) declare that they have no competing interests'.

## Authors' contributions

JÅ and MT designed the study, JÅ operated the animals. MT and JÅ prepared the manuscript and A-KH and PA reviewed. All authors read and approved the final manuscript.

## Pre-publication history

The pre-publication history for this paper can be accessed here:



## Figures and Tables

**Table 1:** Bone volume in the remodelled area and ingrowth distance of new bone into the graft. Bone volume fraction in the remodelled bone measured with Merz grid and expressed in percent of total tissue volume. Bone and vasculature ingrowth distance from living tissue into the graft.

	**Zoledronat**		**Control**		**p**
	Mean	SD	Mean	SD	
New-formed bone (%)	22	7	14	9	0.03
Remaining graft bone (%)	13	6	5	5	0.008
Total Bone (%)	35	0.12	19	0.13	0.01
Bone ingrowth distance into graft (mm)	2,65	0.48	2,12	0.63	0.052 ns
Vasculature ingrowth distance (mm)	4.2	0.90	3.90	1.01	Ns
